# A Children's Paradise

**Published:** 1887-12-24

**Authors:** 


### A Children's Paradise.

Take the contents of half-a-dozen toy-shops ; don't hide any of them away in drawers and boxes, but pile them tier above tier in the most attractive fashion you can imagine ; let the eye rest on dolls great and small, on horses and goats, and all the mystic animals whose aspect is so unlike the picture roused in the adult mind by their names that it gives up in despair the attempt to catalogue them, and accepts with becoming gratitude the courteous explanations vouchsafed by the more imaginative juvenile ; let tops spin through the bewildered brain ; let mechanical toys of every kind speak and sing and move in their conscientious, if somewhat angular and laborious fashion ; let the silver tinkling of musical boxes dominate all ; in a word, let a child's dream of Santa Claus and his gifts be magnified ten times, and then realised to the full—and you will picture in some degree the appearance of one of the largest saloons in Willis's Rooms on the 19th of this month, when the *Truth* toys were exhibited before being sent to their destination—the various children's wards of the London hospitals and workhouses.

### How the Toys came there.

It may be necessary to explain to some of our provincial readers why these toys have thus the honoured name of *Truth* prefixed to them. It may interest all to learn how the fund that supplies this countless throng of playthings arose and grew. In 1880 a brilliant inspiration came to Mr. Labouchere, the intrepid editor of *Truth*. He thought of Christmas-tide, the most joyous season of all the year ; he thought of it as the time when all care, and sadness, and enmity are thrown aside, and when the old cast away the burden of their years and become young again, because it is *par excellence* the children's festival—the feast of the Divine Babe, whose coming into the world was told by the angels as "tidings of great joy"—a joy that is renewed in some degree with the birth of every child who comes from God to gladden the hearts of mortal parents. He thought of it as the festival that crowned with some reflection of the Divine glory all weak and helpless creatures, as the commemoration of an event which made childhood, even poor, neglected, outcast childhood, sacred to men for ever. And thus thinking, his mind wandered from the happy children who strive to guess for weeks before what their Christmas presents will be, and confide to their parents what they would like Santa Claus to bring them, with a sure knowledge that somehow the kindly saint will contrive to gratify their wishes ; it wandered from all these to the children who were poor and unhappy, who had not even childhood's rightful dower of health and happiness, but who tossed on beds of pain without even a mother's hand near to smooth the ruffled pillow or cool the fevered brow ; and he asked himself if it was not possible to bring some part of the Christmas gladness to these also.

### The Competition and the Fund.

The question answered itself almost as soon as it was asked. It is rather strange to think that it is one of the easiest things in the world to make people happier if only it strikes one to try. But that is an idea that doesn't come readily to most of us ; it does not grow naturally in many minds ; it has to be put there. This was what Mr. Labouchere tried to do—to put into the minds of the readers of his journal the idea of bringing a ray of Christmas sunshine to the sick children in London hospitals, by giving to them some of the toys many of them were then making or buying for children they knew. He thought that fathers and mothers would be glad to do it, because those who love their own children truly love all other children for their sake ; and he was quite sure the children themselves would willingly help, because children are your true socialists and humanitarians, and love to picture unknown boys and girls who, in different circumstances, care for just the same toys and games as they do themselves ; and he guessed that besides these there were some people who had not very much Christmas work to do, who were not under the sweet compulsion of giving labour and making sacrifices to add to the happiness of others by some token of love and thoughtfulness. It occurred to Mr. Labouchere that all these might unconsciously co-operate in providing the Christmas gifts he wanted for the sick children who had roused his soul to pity. Therefore, he announced in the journal he edited what he wanted to do, and to encourage people in the task to which he invited them, he offered a prize of four guineas to whoever should produce the best home-made toy. He also asked those who had not time or skill to make toys to send subscriptions for the purchase of some to supplement those of the competitors ; and this was how the fund began.

### How the Fund Grew.

Facts soon demonstrated the accuracy of Mr. Labouchere's estimate of human nature. Toys and subscriptions came in larger numbers than he had anticipated ; and the notion prospered so well, that next year he sent out another invitation, similar to the last, but offering this year prizes to the amount of fifteen guineas. This year the gifts and subscriptions were so large that not a sick child in any of the London hospitals was left without a toy of some sort. Besides this, there being some money in hand beyond what was needed for these personal gifts, the children's wards in several hospitals received gifts of more costly toys, to remain there always for the amusement of the little patients within the walls. Also a great magic lantern was bought for lending to all manner of charitable institutions for the amusement of their inmates. In 1882 the fund grew larger still, and it was possible to extend the kindly work done by it. This year not only sick children but all the children in the London workhouses shared in the distribution of toys. If you think of it, these workhouse children are often more to be pitied than the little hospital patients. The latter are prisoners only for a time, and by the divine right of sickness can claim kindness and consideration while they are in hospital. They are sure of a little petting ; but the workhouse boys and girls grow up in a gray, cold atmosphere, a routine from which affection and gladness are too often banished. It must have been a very strange and new thought to many of those "charity children" that there was some one whom they had never seen, who cared enough for them to send them a gift to brighten one day of their monotonous year. Do you not think that it would help them more than many sermons and much learning of the catechism to realise the infinite love of Some One else whom none of us have yet seen, but in whose care we all believe ? A doll or a humming top seems a very trivial and undignified means to such a great end, but it is wonderful how much more powerful little things often are then big.

### More and More.

In 1883 the Toy Fund was larger than ever, no fewer than 9,000 toys being displayed to an interested crowd of visitors before they were distributed among the hospitals and workhouses. Besides these there was a special gift for the workhouse children. Some kind-hearted person, whose name has never been revealed, sent 5,000 new sixpences to be distributed among them ; and a new, bright, clean sixpence, fresh from the Mint, always seems worth twice as much as one that has been soiled and worn by frequent transit through a hundred hands. To children who are not over familiar with even pennies of their "very own" these sixpences would give a sense of conscious wealth a Rothschild might sometimes envy. Your Crœsus can't really enjoy money ; he is too much used to it ; it is the unfamiliar that we appreciate. This year, too, there began a new set of gifts—not for children this time, but for old people. The old women in the workhouses received packets of tea, the old men small quantities of tobacco and snuff. Of course we all know—at least we have all been told—that the consumption of tobacco in any form may lead to poisoning by nicotine; and quite lately a serious medical journal has included tea-drinking among deadly physiological sins; but, in spite of all this, human nature clings to indulgences which experience has taught it are practically harmless, and we, at least, will not shake our heads over these little concessions to our brethren's harmless weaknesses—which are likewise our own—and are thoroughly glad that these old folks, whose muscles have done so much of life's work that their nerves may safely be left to take care of themselves, have at least once a year these tiny luxuries to warm their aged and slow-flowing blood. It is needless to detail the yearly growth of the *Truth* Toy Fund in all its branches. Suffice it to say that the special workhouse subscription has grown year by year till now, in 1887, the tea prepared for distribution amounts to 222 lbs., which is balanced by 74 lbs. of tobacco and 27 lbs. of snuff; whilst the anonymous donor of the sixpences has increased the number of them to 10,000.

### 10,000 Sixpences!

Sixpence is not in itself a large sum; but when it is multiplied by ten thousand it represents one that is by no means small. Take it arithmetically. Ten thousand sixpences equals five thousand shillings, and this sum, divided by twenty, amounts to £250. That is a good deal for any one person to spend on a Christmas gift, especially to spend on those of whom he has no personal knowledge; but if these 10,000 sixpences bring back ten thousand blessings, besides making ten thousand hearts beat more gladly, the sum they amount to will not be wastefully expended.

### Pyramids of Dolls.

But now for this year's toys. The first impression one receives on entering the room where the toys are exhibited is—dolls. Rows of dolls, heaps of dolls, high-piled, tapering pyramids of dolls! Never in all her professional career, we are sure, did "Miss Jenny Wren, dolls' dressmaker," see such a number of customers gathered together. How many dolls there are it would be rash to say. One lady sends an army of three hundred, no two of whom are costumed just alike; there are several regiments of fifty and many contributions of dozens and half-dozens, besides single dolls which have been dressed by little hands not yet very familiar with holding a needle nor always expert in its use. There are big dolls and small; dolls in every variety of costume, besides dolls for whom the services of Miss Jenny Wren are still required; talking dolls of limited vocabulary; singing dolls, who advertise their qualifications in a way a prima donna would blush to do—wearing upon their breasts a label that announces plainly, "I sing 'God Save the Queen,'" or "I sing 'The Blue Bells of Scotland.'" There are dolls dressed in the latest developments of fashion, and dolls who wear classic and mediæval costume. An occasional anachronism marks these. Cleopatra, for example, wears a cross among her ornaments, a thing which "the serpent of old Nile" would scarcely, we think, have chosen as a jewel. Then there are dolls dressed as Shakespeare's heroines—Ophelia decked with pansies and rosemary; Portia wearing her crimson doctor's gown; and Juliet clad in the traditional white satin. There are peasant dolls of every nation: milkmaids and shepherdesses from east and west, associating *pro tempore* with queens, and court ladies, and mediæval chatelaines, and fairies and ballet-dancers. There are dolls of the sterner sex—gallant soldiers and sailors, and bold huntsmen ready to blow the inspiring horn. There are boy dolls dressed for cricket and boating, and others in the conventional garb of modern life. We regretted to notice, however, that one of the smartest of these was burdened with an umbrella of such a Gampish fashion that it would have broken the heart of any youth who pretended to taste.

### Tops and Cricket Bats.

What these dolls pretend to do the boys do in truth. For them, therefore, the gallant array of cricket bats at the end of the room would have an attraction that would rival even the battledores and tops. Yet these have their charms for the less robust, and smaller ones will delight in the horses and carts. Then there are wonderful Jacks-in-the-box, that peep up unexpectedly from pots of clotted cream or "real home-made jam." There are shops of all kinds to encourage business ideas in the youthful brain; grocers' and butchers' establishments and miniature kitchens for the girls, whose housewifely souls are further delighted with miniature mangles, and tea-sets of brilliant colouring. There are bricks for the architecturally-disposed and drawing-slates for those of artistic tastes; while trains and locomotives help to train the possible railway officials of the future; and Noah's arks give primitive, not perhaps very accurate, instruction in natural history. Besides these, there are scrap-books, picture-books of all sorts, and puzzle-pictures, whose component parts, if our childish recollections do not play us false, have a marvellous faculty for getting lost. But before that fatality occurs, what pleasure can be got out of their combinations!

### Models and Scenes.

Besides these more portable treasures, there are combinations of figures in scenes that show no little artistic skill on the part of the makers, who are all amateurs, and most of whom state in round school-boy or school-girl hand, that they "made it every bit themselves." Among these is a vivid representation of Blue Beard on the point of killing his wife, while Sister Anne looks anxiously from the tower.

In another, representing life a hundred years ago, a lady is being carried home through the snow—it is cotton wool, but to the eye of fancy it is meant for snow—in her sedan-chair, while two small boys in the foreground pepper each other with snowballs in just the fashion of to-day; for though fashions and modes of locomotion change, child nature remains ever the same, and the games and toys which pleased children long ago, and excite them to-day, will gladden those yet unborn just as much a hundred years hence, if the *Truth* Toy Fund goes on as long—and we hope it may.

### Where the Toys go.

Yes, childhood is always much the same; only it is often a problem difficult to solve, when childhood and delight in childish things comes to an end. The grown-up visitors to the *Truth* toys showed quite a youthful delight in the exhibition; noting the details of their merits with the eyes of approving connoisseurs. And if old people are thus pleased, what will the delight of the children be? You might think, perhaps, that such a crowd of playthings could never find owners, that there are too many of them; but listen to this short statement of the number of children who are wondering now what *Truth* will send them this year. In fifty-one London hospitals there are 5,202 children; in thirty-three workhouses 1,660 children; in twenty-three workhouse schools no fewer than 12,260; in twenty-one workhouse infirmaries, where the inmates are both ill and poor, 1,000; and in eight orphanages there are 1,975 children who have no parents to make Christmas bright for them. That makes a total of 22,097 children, who will all be made happier on Christmas morning by the gift of some one of the toys we have told you of. Innumerable as they seem, we are quite sure that they will not be found to be one too many. And may the love and thought that made and collected and will distribute them, be rewarded by glad and grateful remembrances from the sad or lonely or suffering little ones who receive them.

